# Keratoconus cone location influences ocular biomechanical parameters measured by the Ocular Response Analyzer

**DOI:** 10.1186/s40662-023-00371-0

**Published:** 2024-01-03

**Authors:** Phillip T. Yuhas, Maddison M. Fortman, Ashraf M. Mahmoud, Cynthia J. Roberts

**Affiliations:** 1 College of Optometry, The Ohio State University, Columbus, OH USA; 2 Department of Ophthalmology and Visual Sciences, College of Medicine, The Ohio State University, Columbus, OH USA; 3 Department of Biomedical Engineering, College of Engineering, The Ohio State University, Columbus, OH USA

**Keywords:** Corneal hysteresis, Waveform parameters, Corneal topography, Corneal ectasia, Keratoconus match index

## Abstract

**Background:**

Keratoconus is characterized by asymmetry in the biomechanical properties of the cornea, with focal weakness in the area of cone formation. We tested the hypothesis that centrally-measured biomechanical parameters differ between corneas with peripheral cones and corneas with central cones.

**Methods:**

Fifty participants with keratoconus were prospectively recruited. The mean ± standard deviation age was 38 ± 13 years. Axial and tangential corneal topography were analyzed in both eyes, if eligible. Cones in the central 3 mm of the cornea were considered central, and cones outside the central 3 mm were considered peripheral. Each eye was then measured with the Ocular Response Analyzer (ORA) tonometer. T-tests compared differences in ORA-generated waveform parameters between cohorts.

**Results:**

Seventy-eight eyes were analyzed. According to the axial topography maps, 37 eyes had central cones and 41 eyes had peripheral cones. According to the tangential topography maps, 53 eyes had central cones, and 25 eyes had peripheral cones. For the axial-topography algorithm, wave score (WS) was significantly higher in peripheral cones than central cones (inter-cohort difference = 1.27 ± 1.87). Peripheral cones had a significantly higher area of first peak, p1area (1047 ± 1346), area of second peak, p2area (1130 ± 1478), height of first peak, h1 (102 ± 147), and height of second peak, h2 (102 ± 127), than central cones. Corneal hysteresis (CH), width of the first peak, w1, and width of the second peak, w2, did not significantly differ between cohorts. There were similar results for the tangential-topography algorithm, with a significant difference between the cohorts for p1area (855 ± 1389), p2area (860 ± 1531), h1 (81.7 ± 151), and h2 (92.1 ± 131).

**Conclusions:**

Cone location affects the biomechanical response parameters measured under central loading of the cornea. The ORA delivers its air puff to the central cornea, so the fact that h1 and h2 and that p1area and p2area were smaller in the central cone cohort than in the peripheral cone cohort suggests that corneas with central cones are softer or more compliant centrally than corneas with peripheral cones, which is consistent with the location of the pathology. This result is evidence that corneal weakening in keratoconus is focal in nature and is consistent with localized disruption of lamellar orientation.

## Background

Keratoconus is a bilateral corneal ectasia, in which progressive stromal thinning and increased curvature impart a conical shape. Corneal scarring from advanced disease necessitates corneal transplantation, which is costly and may elicit a wide range of post-surgical complications [1]. Corneal cross-linking is a therapeutic procedure that strengthens chemical bonds between adjacent collagen fibrils in the stroma, stabilizing the cornea in most patients [2]. Thus, there is a need to diagnose and monitor keratoconus in its early stages so that corneal cross-linking therapy can be applied at the first signs of disease progression. Keratoconus is typically diagnosed using a combination of corneal topography maps and corneal tomography maps. Diagnostic criteria and keratoconus classification systems that use these tools and others, such as pachymetry, differ in their interpretation of what clinical signs or tomographic features constitute keratoconus [3–5], however, leading to inconsistency in their clinical application [6].

Measurement of the biomechanical response parameters of the cornea has the potential to supplement the traditional means of corneal assessment in keratoconus [7]. Biomechanical assessment involves quantifying the response of the cornea to an applied load, such as an air puff. The Ocular Response Analyzer (ORA; Reichert, Depew, NY, USA) is a commercial non-contact tonometer that measures the deformation response of the cornea to an applied air puff. It uses an electro-optical system to detect the inward and outward applanation events during corneal deformation [8]. Analysis of these events and the pressures at which they occur generates two measurements of intraocular pressure, Goldmann-correlated intraocular pressure and corneal-compensated intraocular pressure. It also produces a viscoelastic metric called corneal hysteresis (CH), which quantifies the ability of the eye to dissipate energy. CH is lower in patients with keratoconus than in healthy controls, but substantial overlap between the groups diminishes the diagnostic potential of this measurement [9]. However, consideration of distinct waveform parameters derived from the plot of the air pressure and of the infrared applanation waveforms improves the ability of the ORA to detect both manifest keratoconus [10] and subclinical keratoconus [11].

The location of the corneal cone apex differs between patients with keratoconus, usually ranging from the central cornea to its inferior quadrants [12, 13]. The exact location of the conical apex has clinical implications, influencing the design of gas-permeable contact lenses [14], the placement of intracorneal ring segments [15], and the location of corneal cross-linking therapy [16]. Moreover, visual acuity recovery after cross-linking therapy may be greater in patients with central cones than in those with peripheral cones [17, 18]. Little is known, however, about how cone location influences the clinical assessment of the biomechanical response of the cornea. That is, given similar stages of disease severity, will the centrally measured biomechanical parameters of a patient with a central cone differ from those of a patient with a peripheral cone? The fact that lamellar disruption in keratoconus is focal in nature [19] supports the hypothesis put forth by Roberts and Dupps that focal, not global, biomechanical weakening is the primary initiating event in the development of keratoconus [20], leading to asymmetry of corneal properties. If this hypothesis is true, then it is reasonable to expect that corneas with central cones will exhibit different biomechanical response parameters than corneas with peripheral cones, since the commercial tonometers that measure corneal biomechanics deliver their loads (i.e., their air puffs) to the central cornea, and they are unable to assess asymmetry of the responses. Thus, the purpose of this study was to test whether cone location influences the measured biomechanical parameters of the cornea, as assessed by the ORA, in keratoconus.

## Methods

This cross-sectional study was conducted at The Ohio State University (OSU). It is part of a larger project that aims to characterize elastic and viscoelastic biomechanical parameters in eyes with keratoconus.

### Participants

Individuals with previously diagnosed keratoconus (ICD-10 code root H18.6) were prospectively recruited from the OSU optometry clinics and from the surrounding community between May 2022 and July 2023. Exclusion criteria were created to limit confounding variables on corneal biomechanical parameters, and they included: (1) corneal diseases other than keratoconus, including but not limited to corneal dystrophies, severe dry eye syndrome, and pterygium; (2) extensive corneal scarring from keratoconus or any other ocular disease (e.g., herpetic eye disease); (3) glaucoma; (4) diabetes mellitus; (5) history of ocular surgery, save for cataract surgery; and (6) use of orthokeratology contact lenses.

### Data collection

First, axial and tangential corneal topography maps were acquired using an E300 corneal topographer (Medmont; Nunawading, Australia). Then, four measurements were taken from each eye using a third generation ORA G3 tonometer.

### Data processing

For all eyes, cone location was defined as the center of a 2 mm circle of maximum curvature, following an objective, published search algorithm, which searches over a defined region of interest to identify a 2 mm area of maximum tangential and axial curvature in diopters [21]. The magnitude of maximal corneal curvature within this area was defined as Cspot. Disease severity was defined as the magnitude of Cspot on the tangential curvature map. Then, eyes were grouped into four cohorts, based on (1) cone location and (2) type of topography map used to identify the location of the cone. For axial topography, the central-cone cohort included cones with the center of Cspot located within a 1.5 mm radius from the center of the cornea (i.e., within the central 3 mm of the cornea), and for the axial peripheral-cone cohort, the center of Cspot was located outside a 1.5 mm radius from the center of the cornea (i.e., outside the central 3 mm of the cornea). For tangential topography, the center of Cspot was located within a 1.5 mm radius from the map center for the central-cone cohort, and outside a 1.5 mm radius from the center of the cornea for the tangential peripheral-cone cohort. Keratometry values (flat meridian, steep meridian, and corneal cylinder) were also recorded from the axial topography map.

Given the known asymmetry in disease severity and in biomechanical parameters between the eyes in keratoconus [22–25], individual eyes of all participants were treated independently. If one eye had a central cone, and the fellow eye had a peripheral cone, then the two eyes were binned into separate cohorts. If both eyes had central cones, or if both eyes had peripheral cones, then the eyes were binned into the same appropriate cohort.

**Fig. 1:**
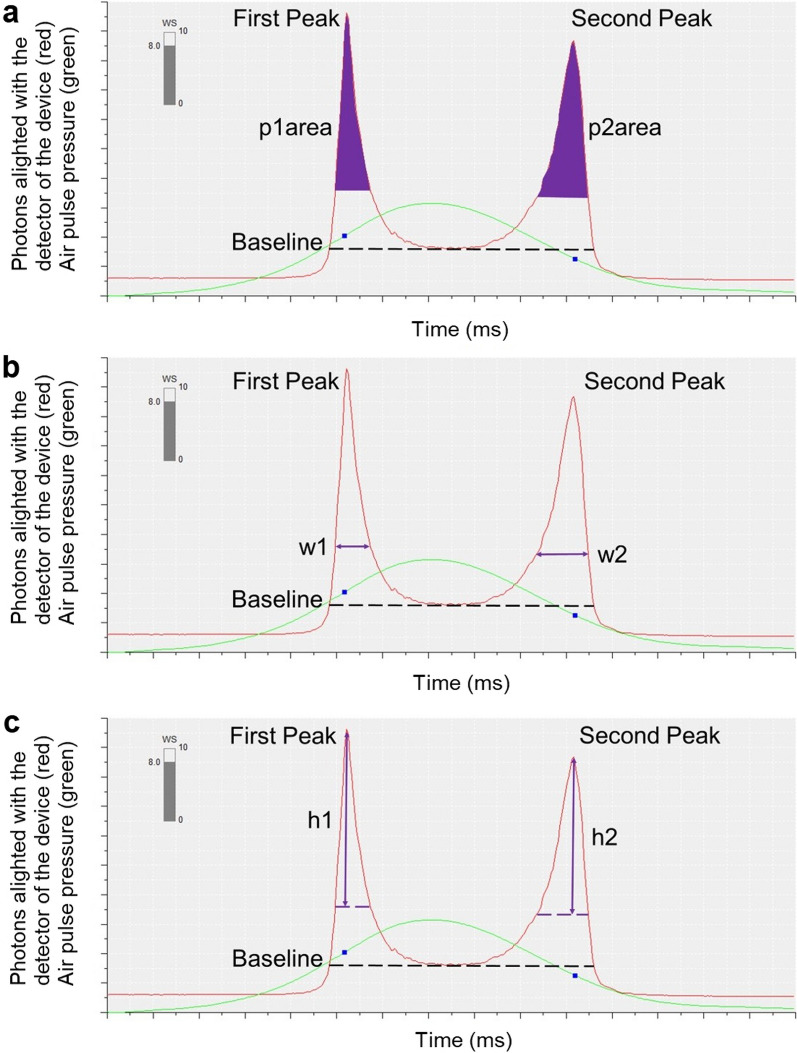
Example waveform parameters. (**a**) Area of the first peak (p1area) and area of the second peak (p2area), (**b**) width of the first peak (w1) and width of the second peak (w2), and (**c**) height of the first peak (h1) and height of the second peak (h2) parameters identified on a representative pressure-applanation waveform generated by the Ocular Response Analyzer. The green traces represent the air pressure delivered to the cornea. The red traces represent the number of photons reflected off the corneal surface and into the infrared light sensor. Peak 1 occurs during first corneal applanation, which happens during inward deformation; and Peak 2 occurs during second corneal applanation, which happens during the outward recovery after cessation of the air puff. The waveform parameters are indicated in purple. The dark blue solid boxes mark the location along the pressure curve where the peaks of the infrared waveform occur. WS is wave score

Data from the ORA measurement with the highest wave score (WS) were analyzed for each eye. In addition to the standard clinical output parameters of waveform score and CH, waveform parameters p1area, p2area, w1, w2, h1, and h2 were exported from the twin-peaked pressure-applanation waveforms generated by the device. Specifically, p1area and p2area represent the area under the curve of the first and second applanation peaks, respectively, from a point 25% above the baseline, which is defined as the nadir of the valley between the two applanation peaks (Fig. 1a); w1 and w2 represent the width of the first and second applanation peaks, respectively, at a point 25% above the baseline (Fig. 1b); and h1 and h2 represent the height of the first and second applanation peaks, respectively, measured from a point 25% above the baseline (Fig. 1c).

### Statistical analysis

Differences in WS, CH, p1area, p2area, w1, w2, h1, and h2 were compared between the central-cone cohort and the peripheral-cone cohort using unpaired t-tests for both curvature algorithms (Statistical Analysis Software; SAS Institute, Cary, NC, USA). Cspot magnitude was compared for tangential topography only; and the steep-meridian keratometry value, the flat-meridian keratometry value, and corneal cylinder were compared for axial topography. Normality was confirmed with the Shapiro–Wilk test. Associations between cone location, as defined both by axial topography and tangential topography, WS, CH, p1area, p2area, w1, w2, h1, and h2 were assessed with linear regression analyses. Very weak associations were defined as r ≤ 0.19, weak associations were defined as r between 0.2 and 0.39, moderate associations were defined as r between 0.4 and 0.59, strong associations were defined as r between 0.6 and 0.79, and very strong associations were defined as r ≥ 0.8. For all statistical analyses, the significance threshold was set at α = 0.05.

## Results

### Demographics

**Fig. 2:**
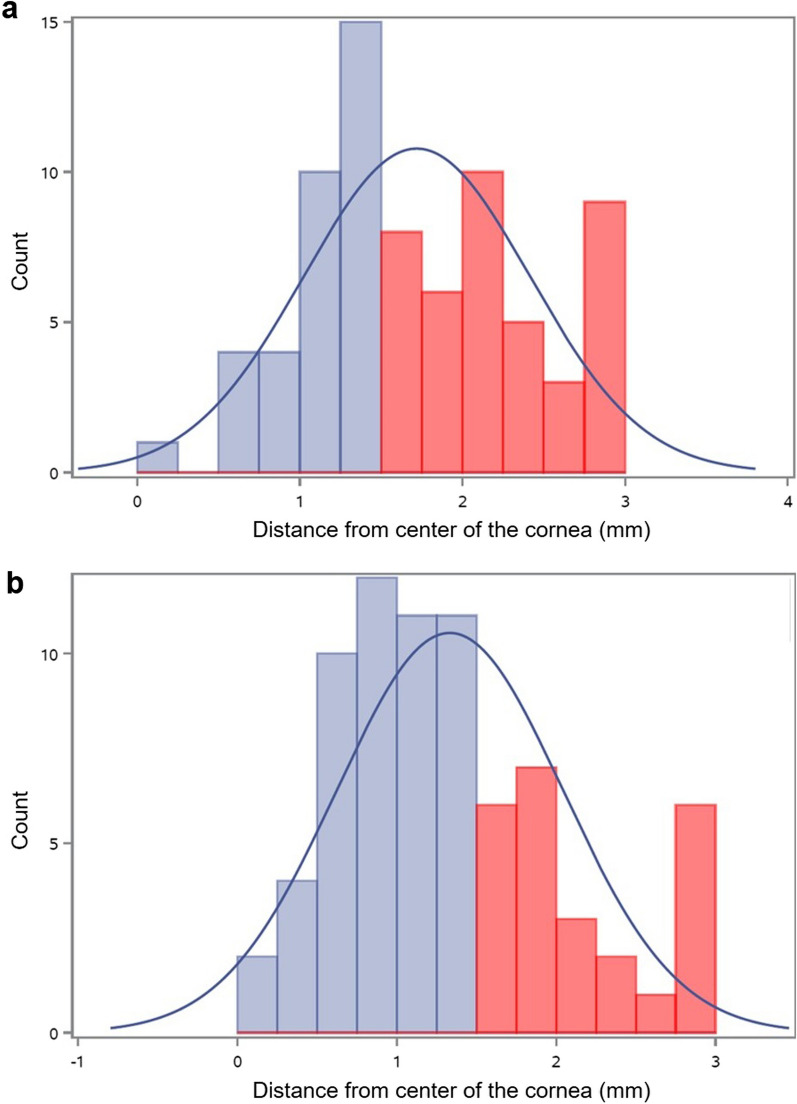
Cone location by cohort. Location of the cone for 78 eyes with keratoconus (**a**) for the axial-topography algorithm and (**b**) for the tangential-topography algorithm. Central cones (light blue) were defined as being within a 1.5 mm radius from the center of the cornea, and peripheral cones (light red) were defined as being outside of a 1.5 mm radius from the center of the cornea. The dark blue line represents the normal distribution curve

In total, 78 eyes of 50 participants (aged 38 ± 13 years; 28% female; 58% White, 32% Black, 6% mixed race, and 4% Hispanic) were analyzed. Figure 2a shows the location of the cones, according to the axial topography maps. For axial topography, 37 eyes had central cones, and 41 eyes had peripheral cones. There was no statistically significant difference in age (*P* = 0.85) between the central-cone cohort (38 ± 14 years) and the peripheral-cone cohort (39 ± 12 years). Figure 2b shows the location of the cones, according to the tangential topography maps. For tangential topography, 53 eyes had central cones, and 25 eyes had peripheral cones. There was no statistically significant difference in age (*P* = 0.10) between the central-cone cohort (37 ± 13 years) and the peripheral-cone cohort (42 ± 14 years).

### Axial-curvature algorithm

#### Keratometry values

The steep-meridian simulated keratometry value, the flat-meridian keratometry value, and corneal cylinder were all significantly greater in the central-cone cohort than in the peripheral-cone cohort (Table 1).

**Table 1:** Differences in keratometry from axial topography and maximum tangential curvature, as defined by Cspot magnitude, between central cones and peripheral cones

Parameters	Central-cone cohort (n = 36)	Peripheral-cone cohort (n = 41)	*P* value
Steep-meridian keratometry value (D)	54.44 ± 10.83	47.42 ± 3.51	0.0006*
Flat-meridian keratometry value (D)	49.90 ± 9.06	44.52 ± 3.18	0.0015*
Corneal cylinder (D)	4.54 ± 3.24	2.90 ± 2.44	0.01*
Maximum tangential curvature (D)	50.93 ± 12.48	53.36 ± 6.22	0.26

Values are mean ± standard deviation. Significance threshold α = 0.05

*P < 0.05, t-test

### Differences in biomechanical parameters between central cones and peripheral cones

Table 2 contains the differences in WS, CH, and waveform parameters between the central-cone and the peripheral-cone cohorts for axial topography. WS, p1area, p2area, h1, and h2 were all significantly lower in the central-cone cohort than in the peripheral-cone cohort. There were no statistically significant differences between the cohorts for CH, w1, and w2.

**Table 2:** Differences in biomechanical parameters between central cones and peripheral cones for axial topography

Parameters	Central-cone cohort (n = 37)	Peripheral-cone cohort (n = 41)	*P* value
Waveform score	5.99 ± 1.96	7.26 ± 1.78	0.004*
Corneal hysteresis (mmHg)	8.90 ± 1.57	9.31 ± 1.60	0.26
P1area	3080 ± 1485	4127 ± 1209	0.001*
P2area	3310 ± 1490	4439 ± 1467	0.001*
W1	18.78 ± 2.46	19.05 ± 2.45	0.64
W2	25.22 ± 6.53	25.12 ± 5.75	0.95
H1	361 ± 167	463 ± 127	0.003*
H2	324 ± 124	426 ± 131	0.0007*

Values are mean ± standard deviation. Significance threshold α = 0.05

*P1area* = area of the first peak; *P2area *= area of the second peak; *W1* = width of the first peak; *W2 *= width of the second peak; *H1* = height of the first peak; *H2* = height of the second peak

*P < 0.05, t-test

### Associations between cone location and biomechanical parameters

**Fig. 3:**
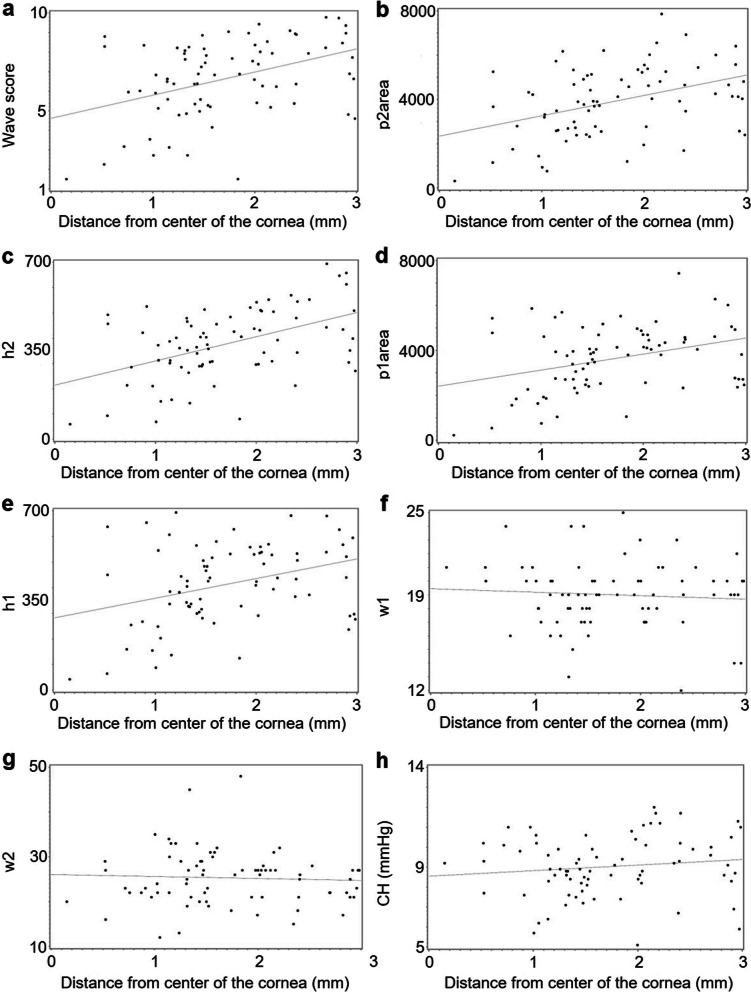
Associations between cone location according to axial topography and biomechanical parameters. Scatter plots showing the relationships between cone location and (**a**) wave score, (**b**) area of the second peak (p2area), (**c**) height of the second peak (h2), (**d**) area of the first peak (p1area), (**e**) height of the first peak (h1), (**f**) width of the first peak (w1), (**g**) width of the second peak (w2), and (**h**) corneal hysteresis (CH). Linear regression lines are solid gray. N = 78 eyes with keratoconus

For axial topography, there were moderate-strength and statistically significant associations between cone location and WS (r = 0.41, *P* = 0.0003; Fig. 3a), between cone location and p2area (r = 0.40, *P* = 0.0003; Fig. 3b), and between cone location and h2 (r = 0.48, *P* = 0.001; Fig. 3c). There were weak but statistically significant associations between cone location and p1area (r = 0.34, *P* = 0.003; Fig. 3d) and between cone location and h1 (r = 0.34, *P* = 0.003; Fig. 3e). There were very weak and non-significant associations between cone location and w1 (r = − 0.07, *P* = 0.53; Fig. 3f), between cone location and w2 (r = − 0.05, *P* = 0.67; Fig. 3g), and between cone location and CH (r = 0.13, *P* = 0.27; Fig. 3h).

### Tangential-curvature algorithm

#### Keratoconus severity

There was no statistically significant difference (*P* = 0.26) in maximum tangential curvature (Table 1), as defined by Cspot magnitude, between the central-cone cohort and the peripheral-cone cohort.

### Differences in biomechanical parameters between central cones and peripheral cones

Table 3 contains the differences in WS, CH, and waveform parameters between central-cone and the peripheral-cone cohorts for tangential topography. WS, p1area, p2area, h1, and h2 were all significantly lower in the central-cone cohort than in the peripheral-cone cohort. There were no significant differences between the cohorts for CH, w1, and w2.

**Table 3:** Differences in biomechanical parameters between central cones and peripheral cones for tangential topography

Parameters	Central-cone cohort (n = 53)	Peripheral-cone cohort (n = 25)	*P* value
Waveform score	6.34 ± 1.95	7.34 ± 1.84	0.04*
Corneal hysteresis (mmHg)	9.02 ± 1.51	9.33 ± 1.76	0.42
P1area	3356 ± 1357	4211 ± 1456	0.01*
P2area	3628 ± 1569	4488 ± 1446	0.02*
W1	18.94 ± 2.46	18.88 ± 2.45	0.92
W2	25.40 ± 6.65	24.68 ± 4.79	0.63
H1	389 ± 154	470 ± 145	0.03*
H2	348 ± 121	440 ± 150	0.005*

Values are mean ± standard deviation. Significance threshold α = 0.05.

*P1area *= area of the first peak; *P2area* = area of the second peak; *W1 *= width of the first peak; *W2* = width of the second peak; *H1 *= height of the first peak; *H2* = height of the second peak

*P < 0.05, t-test

### Associations between cone location and biomechanical parameters

**Fig. 4:**
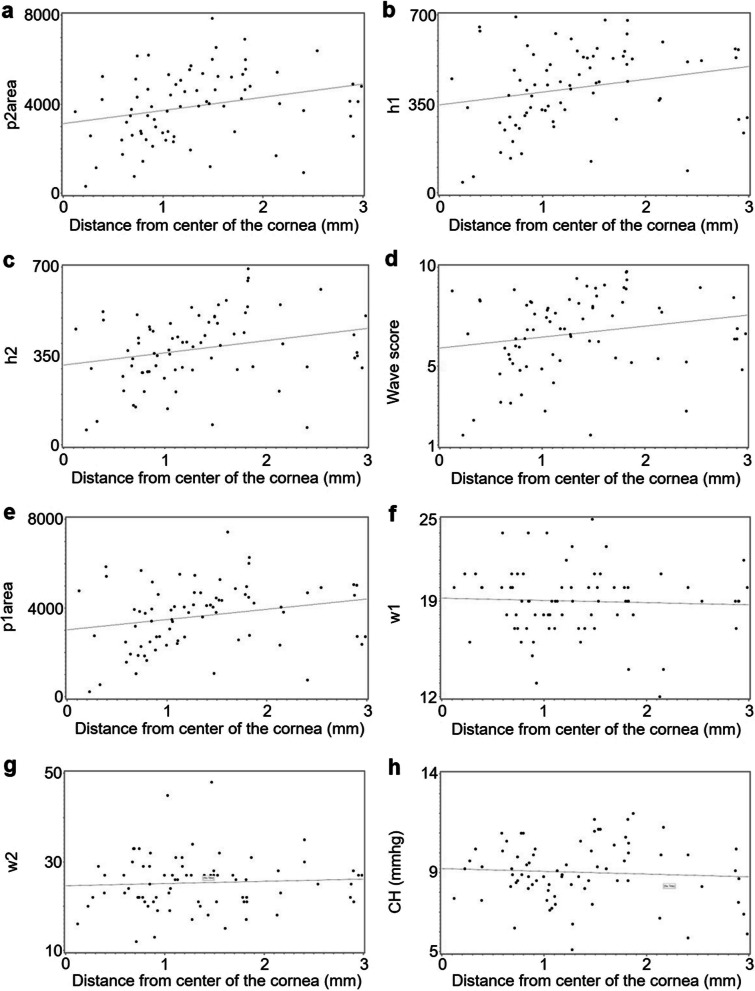
Associations between cone location according to tangential topography and biomechanical parameters. Scatter plots showing the relationships between cone location and (**a**) area of the second peak (p2area), (**b**) height of the first peak (h1), (**c**) height of the second peak (h2), (**d**) wave score, (**e**) area of the first peak (p1area), (**f**) width of the first peak (w1), (**g**) width of the second peak (w2), and (**h**) corneal hysteresis (CH). Linear regression lines are solid gray. N = 78 eyes with keratoconus

For tangential topography, there were weak but statistically significant associations between cone location and p2area (r = 0.27, *P* = 0.02, Fig. 4a), between cone location and h1 (r = 0.23, *P* = 0.05; Fig. 4b), and between cone location and h2 (r = 0.25, *P* = 0.03; Fig. 4c). There were weak and non-significant associations between cone location and WS (r = 0.20, *P* = 0.09; Fig. 4d) and between cone location and p1area (r = 0.23, *P* = 0.05; Fig. 4e). There were very weak and non-significant associations between cone location and w1 (r = − 0.05, *P* = 0.69; Fig. 4f), between cone location and w2 (r = 0.06, *P* = 0.62; Fig. 4g), and between cone location and CH (r = − 0.07, *P* = 0.58; Fig. 4h).

## Discussion

The purpose of this study was to test the hypothesis that cone location in keratoconus influences the measurement of ocular biomechanical parameters with the ORA. We confirmed this hypothesis, as there were significant differences in WS, p1area, p2area, h1, and h2 between the central-cone cohort and the peripheral-cone cohort. The results were similar, regardless of whether the cone was identified using axial topography or using tangential topography. Moreover, there were statistically significant moderate-to-weak associations between cone location and WS, p1area, p2area, h1, and h2 for both curvature algorithms.

The cornea weakens in keratoconus. Uniaxial strip testing of donor tissues first revealed diminished tensile strength in keratoconic corneas compared to healthy corneas [26], and now there is good evidence for elastic deterioration of the cornea in the eyes of patients living with keratoconus [27–29]. One might interpret these results as evidence that the entire cornea weakens in keratoconus, but this interpretation is flawed since none of the methods were able to assess asymmetry. Finite element modeling has identified loss of tissue stiffness, which is localized to a concentric area, as a contributor to keratoconus progression [30], and several groups have used Brillouin microscopy, a form of non-destructive optical elastography [31], to demonstrate focal, not global, weakness in keratoconus that is associated with local corneal thinning and steepening [32–34]. In other words, there is an asymmetry in corneal biomechanical parameters, relative tissue weakness adjacent to relative tissue strength, which is typically not accounted for in their clinical assessment of keratoconus.

**Fig. 5:**
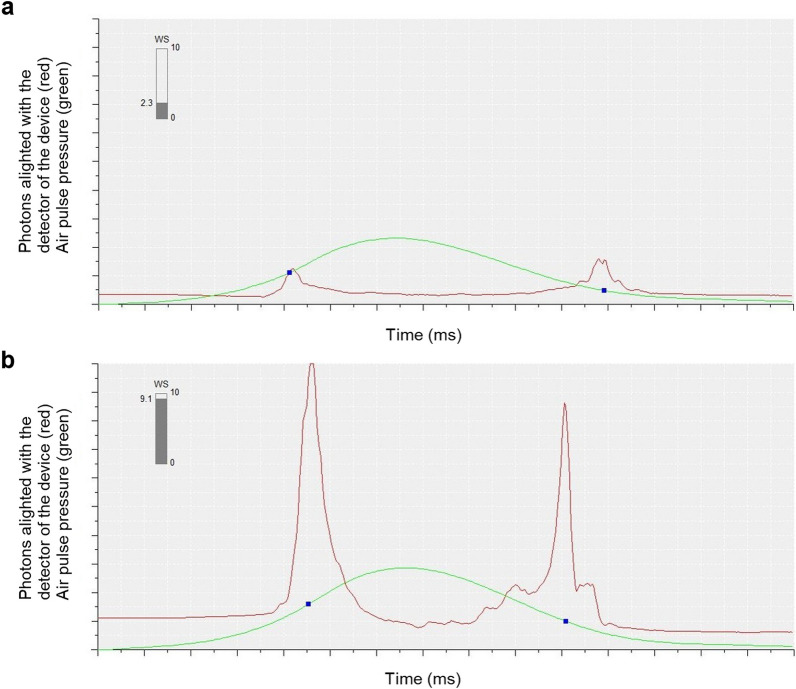
Effect of cone location on waveform peak height. Representative waveforms (**a**) from a participant with a central cone and (**b**) from a participant with a peripheral cone. Note the difference in peak height for both. The green traces represent the air pressure delivered to the cornea. The red traces represent the number of photons reflected off the corneal surface and into the infrared light sensor. The dark blue solid boxes mark the location along the pressure curve where the peaks of the infrared waveform occur. WS is wave score

Our results suggest that clinicians and researchers should consider cone location when interpreting measured biomechanical parameters in eyes with keratoconus. The ORA measures corneal deformation in response to an air puff in an indirect manner. Infrared light is cast onto the cornea, and the light reflecting off the cornea is captured by an infrared sensor. When the cornea applanates, first during the loading phase of the air puff and then again during recovery in the backwards direction, a high number of photons reflecting from the cornea align with the sensor, resulting in a peak on the ORA waveform (see Fig. 1). A large area of applanation allows many photons to reflect into the sensor, resulting in a high peak, and a small area of applanation allows relatively few photons to align with the sensor, resulting in a lower peak. In our results, the central-cone cohort had a smaller p1area and a smaller p2area than the peripheral-cone cohort. This difference in peak areas was driven by the height of the peaks and not by the width of the peaks, as h1 and h2 were significantly smaller in the central-cone cohort than in the peripheral cone cohort, but there were no inter-cohort differences in w1 and w2. These outcomes indicate that the first and second applanation areas were larger for the peripheral-cone cohort than for the central cone-cohort, and we interpret them as strongly suggesting that the peripheral-cone cohort had stiffer central corneas than the central-cone cohort. Consider the analogy of two hollow spheres, sphere A and sphere B. Sphere A and sphere B are filled with the same amount of air and have the same dimensions, but the material of sphere A is stiffer than the material of sphere B. If one were to push on sphere B, it would make a deep but focal indentation. The same force would cause a broad shallow indentation in sphere A, the stiffer sphere. In the same way, an ORA air puff delivered to a relatively stiff central cornea (e.g., peripheral keratoconus) would elicit a boarder applanation area, and therefore a higher waveform peak (Fig. 5), than an ORA air puff delivered to a compliant central cornea (e.g., central keratoconus).

To our knowledge, no other group has reported differences in biomechanical parameters between patients with central keratoconus and patients with peripheral keratoconus using the ORA, possibly due to the limitations of the device. First, the waveform parameters are not displayed in the user interface and must be exported for analysis. Second, as stated above, CH is not a sensitive metric for the detection of keratoconus since it measures viscoelasticity rather than elastic response [9]. The more sensitive keratoconus match index (KMI), based on waveform parameters other than CH [10, 11], is absent from the most recent version of the device (ORA G3).

The need for a new KMI is glaring when the performance of the ORA is compared against that of the cornea visualization with Scheimpflug technology (Corvis ST; Oculus, Wetzlar, Germany) tonometer. The Corvis ST uses a high-speed Scheimpflug camera to capture images of air-puff-induced corneal deformation, which are then analyzed to produce elastic biomechanical parameters of the living human eye [35]. Many studies have demonstrated elastic weakening of the keratoconic cornea using the Corvist ST [27–29, 36], and the device’s biomechanical index, which is a metric that comprises an array of elastic biomechanical parameters, is able to differentiate corneas with keratoconus from healthy corneas with exceptional accuracy [37]. Pertinent to the present study, Bruner and colleagues used the Corvis ST to measure increasing corneal stiffness with increasing cone eccentricity in keratoconus by analyzing elastic outcome measures: stiffness parameter at first applanation, stiffness parameter at highest concavity, deformation amplitude ratio, and integrated inverse radius [38]. These results on the Corvis ST align with ours on the ORA and offer cross-device evidence for the focal nature of the biomechanical weakening in keratoconus.

Despite its limitations, the ORA can still be a useful tool in the assessment of ocular biomechanics. First, given that ORA received approval from the Food and Drug Administration nearly two decades ago, that it is inexpensive, and that it has utility in the assessment of glaucoma risk [39, 40], is more common in primary eye care clinics, where keratoconus is often detected, than the Corvis ST, which most often is found in specialty cornea clinics. Second, the ORA has the potential to measure both the viscoelastic properties of the eye (e.g., CH) and the elastic parameters of the eye, the latter through analysis of the shape of the peaks of the ORA waveform [41]. The Corvis ST can only quantify the elastic parameters of the eye at the current time. Finally, for the purpose of this study, the delivery of an air-puff to the central cornea allows for the quantification of the effect of cone location on biomechanical parameters in patients with keratoconus. Although the ORA and the Corvis ST vary in their assessment of corneal deformation in response to a central air puff, the underlying principle that we support here – namely that of asymmetry in corneal biomechanical parameters in keratoconus – is generalizable across both of them.

Beyond informing our knowledge of corneal weakness in keratoconus, regional variability of the biomechanical parameters has practical implications. First, future clinical studies on corneal biomechanics in keratoconus may consider cone location during study design and during data analysis. A study cohort with a disproportionate number of central cones may skew results toward greater corneal compliance in the disease, compared against a cohort with a disproportionate number of peripheral cones, which may skew results toward greater corneal stiffness. Furthermore, a study cohort with an overrepresentation either of central cones or peripheral cones would likely not be generalizable to a broader population of patients with keratoconus. Rather, a keratoconus study with a mix of central cones and peripheral cones will maximize its applicability. In the clinic, a patient with a peripheral cone may exhibit greater corneal stiffness than a patient with a central cone. In a case of manifest keratoconus, this difference would not influence clinical decision making. However, if the patient had early-stage peripheral keratoconus where there was no clear steepening indicated on the corneal topography map and there were no signs of the disease on slit-lamp biomicroscopy, measurement of biomechanical parameters, as a supplemental means of disease detection, might lead the clinician to conclude that the cornea was relatively stiffer globally without detecting the focal peripheral weakness, potentially delaying timely diagnosis and management, or even misdiagnosing the risk in screening for refractive surgery.

In our population of participants with keratoconus, cone location varied based on topography algorithm. There were more central cones in the tangential topography maps than in the axial topography maps. The difference is expected since the axial curvature algorithm is the mathematical average of the tangential curvature algorithm, so the cone will be represented more peripherally in an axial map than a tangential map. This difference is also consistent with the literature, which reports varying cone location for different map types and for different instruments [13, 42]. It did not have a marked effect on our results, however, as there were significant differences in p1area, p2area, h1, and h2 between the peripheral-cone and central-cone cohorts for both types of curvature. There were also significant associations between cone location and those waveform parameters for both topography curvature algorithms, demonstrating that the further the cone is from the center of the cornea, the larger the magnitude of a given stiffness parameter.

This study has several limitations. First, our sample size was small, and we were unable to detect a difference in CH based on cone location. Beyond the impact that our sample size may have had on our outcomes, this result makes sense because the cornea is not the only ocular structure that influences the ORA waveform. There is evidence that the sclera also contributes, as CH and other waveform parameters of the second peak of the waveform are reduced in eyes that have received a scleral buckle for the repair of retinal detachment, compared to fellow eyes [43]. Thus, CH may be interpreted as ocular hysteresis, rather than as corneal, alone. Second, we did not consider pachymetry values in our analysis. In keratoconus, corneal thinning and corneal steepening occur in conjunction with thinning which drives a steepening response [44], so it is likely that central corneal thickness varied between the central-cone and the peripheral-cone cohorts. According to the biomechanical cycle of decompensation in ectasia [20], both focal thinning and focal steepening result from a focally reduced modulus of elasticity, for which we provide in vivo evidence in this study. Since focal thinning and focal steepening are so intimately connected in keratoconus and other ectasias, only measuring focal steepening, in the form of Cspot, was likely sufficient to study the focal nature of corneal decompensation in keratoconus. Finally, our study was cross-sectional and therefore could not assess how the biomechanical parameters both of central and peripheral cones changed with time. Future studies should consider a longitudinal approach to characterizing the interplay between cone location and corneal biomechanical parameters in order to improve risk profiling for both the detection of keratoconus and for its management.

## Conclusions

We have demonstrated that cone location in keratoconus influences the measurement of biomechanical parameters of the cornea when assessed with the ORA. Specifically, ORA waveform parameters p1area, p2area, h1, and h2 were significantly lower in the central-cone cohort than in the peripheral cone cohort, both for when cone location was determined with axial topography and for when cone location was determined with tangential topography. We interpret our results as suggesting that participants with peripheral cones had stiffer central corneas than participants with central cones. This interpretation is consistent with previous works that suggest localized lamellar disorganization and focal corneal weakening in the disease. It is recommended that researchers and clinicians consider cone location when analyzing biomechanical data.

## Data Availability

The datasets used and/or analyzed during the current study are available from the corresponding author on reasonable request.

## Abbreviations

CH: Corneal hysteresis
Corvis ST: Cornea visualization with Scheimpflug technology
KMI: Keratoconus match index
ORA: Ocular Response Analyzer
OSU: Ohio State University
WS: Wave score

## References

[1] Power BJ, Power WJ, Albert DM, Miller JW, Azar DT, Young LH (2022). Penetrating keratoplasty and complications management. Albert and Jakobiec’s principles and practice of ophthalmology.

[2] Hersh PS, Stulting RD, Muller D, Durrie DS, Rajpal RK, United States Crosslinking Study G (2017). United States multicenter clinical trial of corneal collagen crosslinking for keratoconus treatment. Ophthalmology.

[3] Piñero DP, Nieto JC, Lopez-Miguel A (2012). Characterization of corneal structure in keratoconus. J Cataract Refract Surg.

[4] Belin MW, Duncan JK (2016). Keratoconus: the ABCD Grading System. Klin Monbl Augenheilkd.

[5] Tang M, Li Y, Chamberlain W, Louie DJ, Schallhorn JM, Huang D (2016). Differentiating keratoconus and corneal warpage by analyzing focal change patterns in corneal topography, pachymetry, and epithelial thickness maps. Invest Ophthalmol Vis Sci.

[6] Zhang X, Munir SZ, Sami Karim SA, Munir WM (2021). A review of imaging modalities for detecting early keratoconus. Eye (Lond).

[7] Esporcatte LPG, Salomao MQ, Lopes BT, Vinciguerra P, Vinciguerra R, Roberts C (2020). Biomechanical diagnostics of the cornea. Eye Vis (Lond).

[8] Luce DA (2005). Determining in vivo biomechanical properties of the cornea with an ocular response analyzer. J Cataract Refract Surg.

[9] Fontes BM, Ambrósio R, Velarde GC, Nosé W (2011). Ocular response analyzer measurements in keratoconus with normal central corneal thickness compared with matched normal control eyes. J Refract Surg.

[10] Labiris G, Gatzioufas Z, Sideroudi H, Giarmoukakis A, Kozobolis V, Seitz B (2013). Biomechanical diagnosis of keratoconus: evaluation of the keratoconus match index and the keratoconus match probability. Acta Ophthalmol.

[11] Labiris G, Giarmoukakis A, Gatzioufas Z, Sideroudi H, Kozobolis V, Seitz B (2014). Diagnostic capacity of the keratoconus match index and keratoconus match probability in subclinical keratoconus. J Cataract Refract Surg.

[12] Eliasy A, Abass A, Lopes BT, Vinciguerra R, Zhang H, Vinciguerra P (2020). Characterization of cone size and centre in keratoconic corneas. J R Soc Interface.

[13] Steinwender G, Kollenc A, Shajari M, Sommer M, Borenich A, Horwath-Winter J (2022). Determining the center of a keratoconus: comparison of different tomographic parameters and impact of disease severity. Front Med (Lausanne).

[14] Sorbara L, Dalton K (2010). The use of video-keratoscopy in predicting contact lens parameters for keratoconic fitting. Cont Lens Anterior Eye.

[15] Colin J, Cochener B, Savary G, Malet F (2000). Correcting keratoconus with intracorneal rings. J Cataract Refract Surg.

[16] Greenstein SA, Fry KL, Hersh PS (2012). Effect of topographic cone location on outcomes of corneal collagen cross-linking for keratoconus and corneal ectasia. J Refract Surg.

[17] Shetty R, Nuijts RM, Nicholson M, Sargod K, Jayadev C, Veluri H (2015). Cone location-dependent outcomes after combined topography-guided photorefractive keratectomy and collagen cross-linking. Am J Ophthalmol.

[18] Mimouni M, Sorkin N, Trinh T, Hatch W, Singal N, KEI CXL Study Group (2021). Central versus paracentral cone location and outcomes of accelerated cross-linking in keratoconus patients. Eye (Lond).

[19] Meek KM, Tuft SJ, Huang Y, Gill PS, Hayes S, Newton RH (2005). Changes in collagen orientation and distribution in keratoconus corneas. Invest Ophthalmol Vis Sci.

[20] Roberts CJ, Dupps WJ (2014). Biomechanics of corneal ectasia and biomechanical treatments. J Cataract Refract Surg.

[21] Mahmoud AM, Roberts CJ, Lembach RG, Twa MD, Herderick EE, McMahon TT (2008). CLMI: the cone location and magnitude index. Cornea.

[22] Zadnik K, Steger-May K, Fink BA, Joslin CE, Nichols JJ, Rosenstiel CE (2002). Between-eye asymmetry in keratoconus. Cornea.

[23] Burns DM, Johnston FM, Frazer DG, Patterson C, Jackson AJ (2004). Keratoconus: an analysis of corneal asymmetry. Br J Ophthalmol.

[24] Dienes L, Kránitz K, Juhász E, Gyenes A, Takács A, Miháltz K (2014). Evaluation of intereye corneal asymmetry in patients with keratoconus. A Scheimpflug imaging study. PLoS One.

[25] Xian Y, Zhao Y, Sun L, Zhang X, Ding L, Liu Z (2023). Comparison of bilateral differential characteristics of corneal biomechanics between keratoconus and normal eyes. Front Bioeng Biotechnol.

[26] Andreassen TT, Simonsen AH, Oxlund H (1980). Biomechanical properties of keratoconus and normal corneas. Exp Eye Res.

[27] Bak-Nielsen S, Pedersen IB, Ivarsen A, Hjortdal J (2014). Dynamic Scheimpflug-based assessment of keratoconus and the effects of corneal cross-linking. J Refract Surg.

[28] Tian L, Huang YF, Wang LQ, Bai H, Wang Q, Jiang JJ (2014). Corneal biomechanical assessment using corneal visualization Scheimpflug technology in keratoconic and normal eyes. J Ophthalmol.

[29] Yang K, Xu L, Fan Q, Zhao D, Ren S (2019). Repeatability and comparison of new Corvis ST parameters in normal and keratoconus eyes. Sci Rep.

[30] Falgayrettes N, Patoor E, Cleymand F, Zevering Y, Perone JM (2023). Biomechanics of keratoconus: two numerical studies. PLoS One.

[31] Prevedel R, Diz-Muñoz A, Ruocco G, Antonacci G (2019). Brillouin microscopy: an emerging tool for mechanobiology. Nat Methods.

[32] Scarcelli G, Besner S, Pineda R, Yun SH (2014). Biomechanical characterization of keratoconus corneas ex vivo with Brillouin microscopy. Invest Ophthalmol Vis Sci.

[33] Scarcelli G, Besner S, Pineda R, Kalout P, Yun SH (2015). In vivo biomechanical mapping of normal and keratoconus corneas. JAMA Ophthalmol.

[34] Shao P, Eltony AM, Seiler TG, Tavakol B, Pineda R, Koller T (2019). Spatially-resolved Brillouin spectroscopy reveals biomechanical abnormalities in mild to advanced keratoconus in vivo. Sci Rep.

[35] Ambrosio R, Ramos I, Luz A, Faria FC, Steinmueller A, Krug M (2013). Dynamic ultra high speed Scheimpflug imaging for assessing corneal biomechanical properties. Rev Bras Oftalmol.

[36] Sedaghat MR, Momeni-Moghaddam H, Ehsaei A, Vinciguerra R, Zamani O, Robabi H (2023). Comparison of corneal biomechanical properties in healthy thin corneas with matched keratoconus eyes. J Cataract Refract Surg.

[37] Vinciguerra R, Ambrósio R, Elsheikh A, Roberts CJ, Lopes B, Morenghi E (2016). Detection of keratoconus with a new biomechanical index. J Refract Surg.

[38] Bruner C, Mahmoud A, Roberts CJ (2022). Cone location and corneal stiffness in keratoconus. Invest Ophthalmol Vis Sci.

[39] Susanna CN, Diniz-Filho A, Daga FB, Susanna BN, Zhu F, Ogata NG (2018). A prospective longitudinal study to investigate corneal hysteresis as a risk factor for predicting development of glaucoma. Am J Ophthalmol.

[40] Medeiros FA, Meira-Freitas D, Lisboa R, Kuang TM, Zangwill LM, Weinreb RN (2013). Corneal hysteresis as a risk factor for glaucoma progression: a prospective longitudinal study. Ophthalmology.

[41] Qin X, Yu M, Zhang H, Chen X, Li L (2019). The mechanical interpretation of ocular response analyzer parameters. Biomed Res Int.

[42] Sedaghat MR, Momeni-Moghaddam H, Azimi Khorasani A, Belin MW, Monfared N, Wolffsohn JS (2021). Comparison of keratoconus cone location of different topo/tomographical parameters. Curr Eye Res.

[43] Taroni L, Bernabei F, Pellegrini M, Roda M, Toschi PG, Mahmoud AM (2020). Corneal biomechanical response alteration after scleral buckling Surgery for rhegmatogenous retinal detachment. Am J Ophthalmol.

[44] Roberts CJ, Knoll KM, Mahmoud AM, Hendershot AJ, Yuhas PT (2023). Corneal stress distribution evolves from thickness-driven in normal corneas to curvature-driven with progression in keratoconus. Ophthalmol Sci.

